# Mast Cell Cytokines IL-1, IL-33, and IL-36 Mediate Skin Inflammation in Psoriasis: A Novel Therapeutic Approach with the Anti-Inflammatory Cytokines IL-37, IL-38, and IL-1Ra

**DOI:** 10.3390/ijms22158076

**Published:** 2021-07-28

**Authors:** Pio Conti, Fabrizio E. Pregliasco, Rosa G. Bellomo, Carla E. Gallenga, Alessandro Caraffa, Spyros K. Kritas, Dorina Lauritano, Gianpaolo Ronconi

**Affiliations:** 1 Postgraduate Medical School, University of Chieti, 66100 Chieti, Italy; 2 Istituto Ortopedico Galeazzi, 20100 Milano, Italy; fabrizio.pregliasco@unimi.it; 3 Facoltà di Scienze dell’Educazione Motoria, Università “Carlo Bo”, 61029 Urbino, Italy; rosa.bellomo@uniurb.it; 4 Department of Biomedical Sciences and Specialist Surgery, University of Ferrara, 44100 Ferrara, Italy; gllcln@unife.it; 5 School of Pharmacy, University of Camerino, 62032 Camerino, Italy; alecaraffa@libero.it; 6 Department of Microbiology and Infectious Diseases, Aristotle University of Thessaloniki, 54250 Macedonia, Greece; skritas@vet.auth.gr; 7 Medicine and Surgery Centre of Neuroscience of Milan, University of Milan-Bicocca, 20100 Milano, Italy; dorina.lauritano@unimib.it; 8 Clinica dei Pazienti del Territorio, Fondazione Policlinico Gemelli, 00168 Rome, Italy; gianpaolo.ronconi@policlinicogemelli.it

**Keywords:** psoriasis, cytokine, mast cell, IL-1, inflammation, immunology, IL-37, IL-38, IL-1Ra

## Abstract

Psoriasis (PS) is a skin disease with autoimmune features mediated by immune cells, which typically presents inflammatory erythematous plaques, and is associated with many comorbidities. PS exhibits excessive keratinocyte proliferation, and a high number of immune cells, including macrophages, neutrophils, Th1 and Th17 lymphocytes, and mast cells (MCs). MCs are of hematopoietic origin, derived from bone marrow cells, which migrate, mature, and reside in vascularized tissues. They can be activated by antigen-provoking overexpression of proinflammatory cytokines, and release a number of mediators including interleukin (IL)-1 and IL-33. IL-1, released by activated keratinocytes and MCs, stimulates skin macrophages to release IL-36—a powerful proinflammatory IL-1 family member. IL-36 mediates both innate and adaptive immunity, including chronic proinflammatory diseases such as psoriasis. Suppression of IL-36 could result in a dramatic improvement in the treatment of psoriasis. IL-36 is inhibited by IL-36Ra, which binds to IL-36 receptor ligands, but suppression can also occur by binding IL-38 to the IL-36 receptor (IL-36R). IL-38 specifically binds only to IL-36R, and inhibits human mononuclear cells stimulated with IL-36 in vitro, sharing the effect with IL-36Ra. Here, we report that inflammation in psoriasis is mediated by IL-1 generated by MCs—a process that activates macrophages to secrete proinflammatory IL-36 inhibited by IL-38. IL-37 belongs to the IL-1 family, and broadly suppresses innate inflammation via IL-1 inhibition. IL-37, in murine models of inflammatory arthritis, causes the suppression of joint inflammation through the inhibition of IL-1. Therefore, it is pertinent to think that IL-37 can play an inhibitory role in inflammatory psoriasis. In this article, we confirm that IL-38 and IL-37 cytokines emerge as inhibitors of inflammation in psoriasis, and hold promise as an innovative therapeutic tool.

## 1. Introduction

Approximately 3% of the population of the United States of America and 2% of the global population suffer from psoriasis. This disease is associated with a high degree of morbidity, and the patients affected have a decreased quality of life. Psoriasis is a common disease that presents skin lesions, dysregulated immune system, and chronic inflammation—problems that are still unresolved [1], although in recent years, biomedical research has made substantial advances in elucidating the pathogenic mechanisms. Predisposition to psoriasis may be due to environmental factors such as stress, drugs, microorganisms, smoking, and trauma, but also to the dysregulation of the immune system and, in particular, to the imbalance of some immunoregulatory cytokines [2]. Psoriasis is a disease believed to be of autoimmune nature, which causes scaly patches on the skin and redness in the lesions, due to the increased numbers of capillaries. It has previously been reported in murine models that psoriatic skin can be xenografted onto immunodeficient mice, transferring the disease [3]. Psoriasis typically affects the scalp, elbows, sacral region, and knees, but it can also occur throughout the body [4]. In particular, patients may present thickening of the epidermis, leukocyte infiltration, and parakeratosis [5]. In the skin, cytokines produced by activated immune cells contribute to the induction of psoriasis [6]. The disease involves the immune cells—including macrophages, mast cells (MCs), neutrophils, and Th1, Th17, and Th22 lymphocytes—which contribute to epidermal proliferation and production of cytokines and chemokines [7]. In addition, around the capillaries of the dermis and epidermis, T CD3^+^ and T CD8^+^ lymphocytes and CD11c^+^ dendritic cells can be detected, confirming the immunological intervention in this disease [8]. Moreover, studies have indicated that the numbers of MCs are increased in psoriatic skin, while the numbers of regulatory T (Treg) cells are not altered [9]. In fact, mice deficient in Treg cells develop a skin disease similar to psoriasis, and show a decrease in IL-10 levels [10].

**Figure 1:**
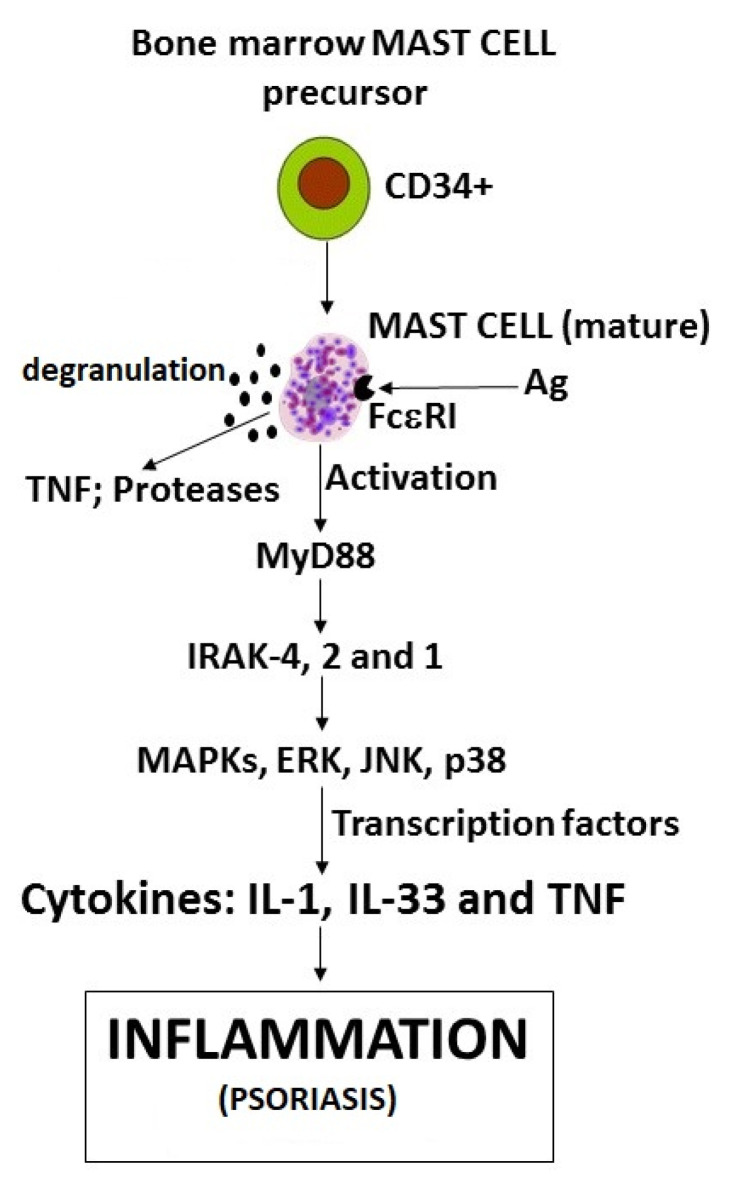
Generation of proinflammatory cytokines by IL-1, IL-33, and TNF from activated mast cells (biochemical pathway), and release of tryptase and TNF by degranulation.

In psoriasis, since keratinocytes produce IL-1, they can be considered to be cells participating in innate immunity that respond early to external insults [11]. Dendritic cells—key sentinels of the immune system—are also increased in psoriatic lesions, and favor the production of Th1 cells and secretion of proinflammatory cytokines and nitric oxide [12]. Dendritic cells, macrophages, and MCs are activated through Toll-like receptors (TLRs), leading to cytokine/chemokine production and induction of inflammation [13]. The cytokines IL-1, tumor necrosis factor (TNF), and IL-6 are secreted at the site of the lesion by keratinocytes. These cytokines activate dendritic cells which, in turn, produce cytokines IL-12 and IL-23, which lead to the differentiation of TH1 and TH17 cells. Moreover, keratinocytes and MCs secrete IL-1, IL-6, TNF, IL-17, and IL-22, and also the chemokines CXCL8, -9, -10, -11, and -20, which participate in inflammatory activity [14] (Figure 1). It is likely that blocking one of these steps above may result in a relief of psoriasis [15].

## 2. Description and Function of Mast Cells (MCs)

It is known that MCs play an important role in the pathophysiology of skin diseases [16]; however, the experiments that best describe the characteristics of MCs in immune and inflammatory processes mainly concern those carried out on mice. This was possible because MC-deficient mutant mice were created, offering the opportunity to study immune and inflammatory processes [17]. Immune cells—such as lymphocytes and macrophages—have subsets, but this is not the case for granulocytes such as MCs, although some authors have divided MCs into subsets MC1 (anti-tumorigenic) and MC2 (pro-tumorigenic) [18]. In addition, it is likely that there are different types of MCs with different functions related to their maturation, but this hypothesis has yet to be confirmed, and is being studied in our laboratory.

MCs are of hematopoietic origin, derived from bone marrow cells, and reside in vascularized tissues [19]; they are sources of biologically active compounds—including cytokines—and arachidonic acid products (prostaglandins and leukotrienes), which mediate inflammatory disorders [20]. IgE-dependent MC activation is a key reaction in allergic diseases and anaphylaxis [21]. Therefore, MCs are activated by high-affinity IgE that binds to the FceRI receptor and leads to degranulation and immediate release (a few seconds) of preformed chemical mediators and TNF from the granules [22] (Table 1). Subsequently (after several hours), there is a de novo synthesis of cytokines and chemokines. MCs intervene in many types of innate or adaptive immune responses [23] (Figure 1). Macrophages and MCs generate caspase-1 and chymase, activate immature pro-IL-1 beta, and transform it into mature IL-1 beta, which amplifies the inflammatory state [24]. The production of IL-1 by MCs increases the number of infiltrating neutrophils with the release of proteases and rising inflammation [25]. TNF is another highly inflammatory cytokine released by activated macrophages, and many other cells, including MCs, which contain preformed TNF that can be released rapidly (after about 10 min) from the cell granules after activation by anti-IgE, or exposure to substance P (SP) or ultraviolet rays (as occurs in the skin) [26]. TNF can also be produced through the synthetic pathway, which requires several hours. MCs can be recruited into inflamed skin by diverse chemotactic molecules (including Vascular endothelial growth factor (VEGF), stem cell factor (SCF), and a number of CC and CXC chemokines) produced by immune cells [27]. MCs have been proposed to influence many biological processes, including autoimmune disorders [28].

## 3. Relationship between Mast Cells and Proinflammatory and Anti-Inflammatory Cytokines in Inflammation

Mast cells are ubiquitous and, when activated, they produce proinflammatory cytokines. In psoriasis, MCs can be activated by IL-1, causing inflammation and triggering a cytokine network including IL-33 and TNF cytokines. Hence, MCs in psoriasis actively participate in the inflammation of blood vessels and skin. In this skin disease, TNF provokes signals and transcripts via the Janus kinase pathway, JAK-STATs (signal transducers and activators of transcription), and NF-κB, producing cytokines that mediate inflammation [29]. On the other hand, MCs produce IL-10, which inhibits hypersensitivity reactions and skin responses to ultraviolet irradiation, dampening inflammation [30]. In addition, MCs produce IL-33 (and are activated by it), which is expressed by skin keratinocytes and endothelial cells, and amplifies the inflammatory process [26]. For instance, another cytokine that plays an important role in psoriasis and participates in the complex cytokine system is IL-36 [31]. The proinflammatory network of cytokines involving MCs in the skin include, as key mediators, IL-33 and IL-36; therefore, inhibiting IL-36 with IL-38 could alleviate the inflammatory state exerted by MCs [32]. In contrast, MCs also perform a protective function of the skin against bacterial infections, by producing some cytokines such as IL-6 that contribute to the killing of infectious bacteria [33]. IL-36 was discovered in 1999, and is a member of the IL-1 family [34]. This family comprises 11 proteins, of which 7 show agonistic activity—IL-1 alpha, IL-1 beta, IL-18, IL-33, IL-36 alpha, IL-36 beta, and IL-36 gamma—3 IL-1Ra, IL36Ra, and IL-38 receptor antagonists, and 1 anti-inflammatory cytokine, IL-37, which translocates to the nucleus and suppresses the transcription of proinflammatory genes [35]. Moreover, IL-37 is secreted intracellularly, where it binds to the receptor IL-18Ra, recruits the TIR8 signal, and suppresses NF-κB and MAPK, with consequent inhibition of inflammation [36]. IL-36 binds to its receptor, called IL-36Ra (IL-36 receptor antagonist), which binds to IL-36R (also called IL-1R6) but does not recruit the co-receptor and, consequently, does not transmit the signal [37]. IL-36Ra inhibits the activation of the IL-36 receptor IL-36R signaling pathway, exerting an anti-inflammatory action [38]. All subtypes of IL-36 have a molecular weight of about 18 KDa that binds to the same receptor, also formerly called IL-1RL2 or IL- 1Rrp2 [39]. IL-36 intervenes in both innate and adaptive immunity. This cytokine activates the MAPK and NF-κB pathways, and induces the production of proinflammatory cytokines [40]. The binding of IL-36 to its own receptor and to the IL-RAcP co-receptor leads to transduction of the NF-κB signal, or MAPK activation [38]. IL-36 is expressed by macrophages, CD4^+^ T cells, dendritic cells, and other cells [38]. IL-36Ra shares 52% of its homology with IL-1Ra—another cytokine inhibitor of the IL-1 family [31]. IL-36 contributes to the maturation of dendritic cells by stimulating the secretion of IL-12, which participates in the differentiation of T cells in Th1 cells [41]. In addition, IL-36 plays a critical role in Th1 response, and is expressed on the skin as a proinflammatory cytokine in psoriasis [42]. This cytokine has biological activity, including its inflammatory properties on the skin, where it is expressed abundantly by keratinocytes [43]. In psoriatic skin, IL-36 is related to the Th1 and Th17 cytokines, and mediates various autoimmune inflammatory diseases, such as systemic lupus erythematosus, inflammatory bowel disease, ulcerative colitis, Crohn’s disease, and psoriatic arthritis, as well as inflammation of the tissues induced by microbial infections [44]. Therefore, the skin gamma delta T cells Th1 and Th17 produce IL-36, which plays a key role in the pathogenesis of psoriasis. In in vivo experiments, transgenic mice expressing a high amount of IL-36 in cutaneous keratinocytes showed skin lesions similar to psoriasis, demonstrating the importance of this proinflammatory cytokine [45]. In addition, stimulation of IL-36 can also induce a high production of other proinflammatory cytokines, such as IL-6, IL-8, TNF, and a number of chemokines [46,47]. Chemokines are chemoattractant cytokines of leukocyte trafficking to sites of inflammation, though their biological role is not limited to chemotaxis, since they present many biological effects [48]. In fact, chemokines are involved in hematopoiesis, innate and acquired immunity, angiogenesis, and metastases [49]. In in vitro study, MCs stimulated by SP and treated with IL-37 [50,51]—a cytokine with properties similar to those of IL-38—do not inhibit chemokines CC5 or CXCL8 (which attract mononuclear cells and neutrophils, respectively) (unpublished data), while IL-38 does. Suppressing IL-36—for example with IL-38 binding to IL-36R—would mean providing safe relief to the patients with chronic proinflammatory diseases, including psoriasis. To date, many treatments for psoriasis have been proposed—such as bone marrow transplantation, drug treatments, or anti-TNF therapy—but all still need to be improved. Moreover, some antibodies—such as anti-IL-17, anti-IL-17 receptor, anti-IL-12/23p40, and anti-IL-23p19—have dramatically changed the treatment of psoriasis. Furthermore, studies on transgenic mice have provided valuable information on the pathogenesis of this disease. Thus, here, we propose new therapy based on the inhibition of inflammatory cytokines produced by mast cells, with the hope that it will have an effective impact on psoriasis—a chronic proinflammatory disease [52].

## 4. IL-33 Is a Proinflammatory Cytokine

IL-33—also called “alarmin”—is one of the danger-associated molecular pattern (DAMP) molecules, also known as endogenous danger signals, with properties to mediate sterile inflammatory responses due to trauma, without the intervention of microorganisms [53]. IL-33 can be released by MCs after physiological stress, as occurs in psoriasis—a non-allergic, hyper-proliferative inflammatory skin disease with an important neurogenic component. MCs, through the production of IL-33, intervene in metabolic homeostasis, and in various diseases of the central nervous system—including stress, which exacerbates psoriasis [54]. In recent years, it has been seen that IL-33, in addition to being involved in the activation of MCs, is also able to stimulate type 2 innate lymphoid cells (ILC2s), Tregs, natural killer cells, and CD8 + T lymphocytes [55]. IL-33 possesses pleiotropic activity in immune responses and, through its ST2 receptor, activates the JNK signaling pathway, demonstrating that by manipulating the ST2/IL-33 ratio one could obtain a better understanding of inflammatory mechanisms. IL-33 is localized in the cell nucleus and, by binding of NF-κB to p65, it can also exert an inhibitory action on the transcription activity of NF-κB; in fact, IL-33, in regulating Th2 cells, shows a contrast to proinflammatory IL-18 [53]. In psoriasis there can be an augmentation of neurotransmitters, such as substance P (SP), which induces VEGF in human mast cells—an effect that leads to increased IL-33 and inflammation [1]. IL-33, which is released from MCs without cleavage by caspase-1, is a critical player in immune response to tissue suffering, and acts through its ST2 receptor—a member of the IL-1 family, located in the human chromosome 2 locus, implicated in T helper 2 (Th2) responses in MC-driven allergic diseases. About 10 years ago, we reported that the neurotransmitter SP induces gene expression and VEGF secretion in human umbilical cord blood-derived cultured mast cells (hCBMCs), and an increased effect with IL-33 administration, involving PKC (protein kinase C) and the MAPK ERK and JNK pathways. These results demonstrate that IL-33 intervenes in inflammatory conditions, including psoriasis, where neurotransmitters can aggravate inflammation. In addition, we have shown that IL-33 administered in combination with SP strongly increases the gene expression of TNF in human mast cells in vitro—an effect that would also occur in psoriasis mediated by neurotransmitters. The activation of MCs by IL-33 causes the release of histamine and cytokines/chemokines, which are very important in rheumatic diseases, including psoriasis.

Taken together, all of the concepts expressed herein demonstrate that IL-33 (“alarmin”) can exacerbate inflammatory allergic skin reaction and increase TH1 response—as occurs in experimental arthritis induced in mice—but also that IL-33 is capable of inhibiting proinflammatory IL-18 released by macrophages, demonstrating anti-inflammatory activity. However, these data are still under study, and remain to be clarified.

## 5. Intervention of IL-1 in Psoriatic Inflammation

Psoriasis is characterized by the activation of MCs, proliferation of keratinocytes, and production of IL-1, and is the result of inflammation [56]. Psoriasis is also induced and/or exacerbated by stress, with the release of proinflammatory neurotransmitters that activate MCs in inflammation.

About 35 years ago, our group pointed out that IL-1 plays an important role in the pathogenesis of many immunological, inflammatory, infectious, and neoplastic diseases [57]. Today, the role of IL-1 has been sufficiently well defined, and it has been shown that this cytokine is strongly linked to inflammation, as a primary function, but also to cellular resistance to infections and foreign antigens [57].

The IL-1 family has 11 cytokine members and 10 receptors, all participating in both immunological and inflammatory responses [58]. IL-1 is a polypeptide whose genes can be activated by various inflammatory perturbations in any tissue or organ, causing the stimulation of a number of cells, including MCs. The release of allergic mediators by MCs is affected by IL-1 and other cytokines. Immune cells—such as macrophages and granulocytes activated by IL-1—can release arachidonic acid metabolites and synthesize proteases, as occurs in PGD2 released by MCs. Furthermore, it has been reported that pro-IL-1, mature IL-1, and TNF can cause a release of histamine from basophilic cells—an effect correlated with the anti-IgE response [59]. Allergens in the skin can activate MCs, which cause the release of acute-phase proteins and cytokines, which can be also synthesized by other activated immune cells, consequently causing intense inflammation. In psoriasis, high levels of histamine can occur during both the acute and late phases, but also the levels of tryptase and PGD2 can be higher. In light of these data, it is pertinent to think that preventing the generation of IL-1 through the blockade of its receptor (IL-1Ra) can have an effective therapeutic response for both psoriasis and other inflammatory diseases.

## 6. IL-1 Receptor Antagonist in Psoriasis

MCs are effector cells in many biological responses, providing an important biological function involving many organs, including the skin [60]. Among the skin diseases, we find psoriasis, which is a chronic relapsing inflammatory disease with several symptoms, including inflammation and immune imbalance.

The disease presents clinical points mediated by inflammatory cytokines, including IL-1, which plays an important proinflammatory role through its IL-1R1 receptor [38]. The IL-1 receptor is expressed in almost all tissues, and its activation causes inflammation; therefore, by blocking this receptor, a therapeutic effect can be obtained in inflammatory diseases mediated by IL-1.

IL-1Ra is found in the bloodstream of healthy subjects at a concentration ranging from 100 to 300 ng/mL, and is a natural molecule of the IL-1 family, most currently used for the treatment of rheumatoid arthritis, but has also been shown to be effective in a broad spectrum of inflammatory states, including heart disease and some autoimmune diseases, as well as having been identified as a candidate stroke drug [61,62]. Serum levels of IL-1Ra are high in many inflammatory and autoimmune diseases, including psoriasis—an effect due to the body’s response to inflammatory stimuli. The IL-1 genes and their proximity to IL-Ra indicate that these two cytokines are very similar, and have the same origin. IL-1Ra binds, at high affinity, to the IL-1R receptor, without inducing the IL-1RAcP co-receptor, and therefore does not cause any change, but performs an antagonistic action by competing with IL-1. IL-Ra has an anti-inflammatory action that limits the production of IL-1 in rheumatoid arthritis and psoriatic conditions, although this inhibition does not completely resolve the pathological clinical signs.

As reported above, IL-1 stimulates the production of IL-6 in MCs—a specific effect, without the implication of degranulation. IL-1Ra can be highlighted in neurological diseases where IL-1, in stimulating the production of IL-6, increases the inflammatory process, but can be also neuroprotective. IL-1Ra administration in rodents has been reported to cross the blood–brain barrier, reducing inflammatory neurological diseases [63]. As IL-1 is implicated in clinical inflammatory brain manifestations, targeting IL-1 can cure related symptoms, including those caused by stress that can exacerbate inflammation in psoriasis.

In inflammatory processes, including psoriasis, IL-1Ra can inhibit inflammation by reducing granulocyte infiltration and releasing proteases, IL-6, and fibrinogen [64]. Treatment with IL-1Ra in arthritis improves pain and itching, and has beneficial effects, with therapeutic results that can also occur in other conditions, including fever, arthralgia, fatigue, rash, and other symptoms related to inflammation [65]. Today, hundreds of thousands of patients worldwide with autoimmune diseases, including psoriasis, are treated with anti-inflammatory cytokines that inhibit IL-1, although a small percentage of those treated may develop bacterial infections. Several data report that IL-1Ra not only relieves the stress caused by IL-1, which aggravates the psychiatric state, but also limits fever and shock—pathological conditions that can affect the health of patients. IL-1Ra deficiency can trigger inflammatory diseases such as arthritis and psoriasis. In in vivo models, rodents with low levels of IL-1Ra can develop a psoriasis-like rash, arthritis, and other diseases related to immunodeficiency, such as tumors and infections with skin pustules and vasculitis [66]. Endothelial cells of the dermis express IL-1R which, after antigen activation, releases IL-1. This potent cytokine stimulates nearby MCs to produce TNF, which has an inflammatory effect and stimulates endothelial cells to produce other proinflammatory compounds, such as chemokines CXCL8, CCL20, and CXCL1, and cytokine IL-6 [67]. In addition, expression of adhesion molecules—including ICAM-1 (intracellular adhesion molecule) and V-CAM (vascular cell adhesion molecule)—can occur at the site of psoriatic inflammation, causing an increase in the number of T cells in loco, which also participate in the inflammatory network. All of these effects can certainly increase the inflammatory state in psoriasis. Using IL-1Ra, the above-mentioned effects can be reduced through IL-1 blocking and, thus, inhibition of inflammation. The satisfactory clinical results of IL-Ra led to the development of the drug anakinra, which has been approved by the Food and Drug Administration (FDA) for the treatment of rheumatoid arthritis.

In conclusion, this article focused on the contribution of MCs and their secreted and released products to psoriatic inflammation, demonstrating that these cells are very important in aggravating the disease—a process that can be limited with the use of new anti-inflammatory cytokines such as IL-37, IL-38, or IL-1Ra. However, in order to verify these effects, improve them, and ascertain their validity, further experiments are needed in the future.

## 7. IL-38: The New Cytokine That Inhibits Inflammation

**Figure 2:**
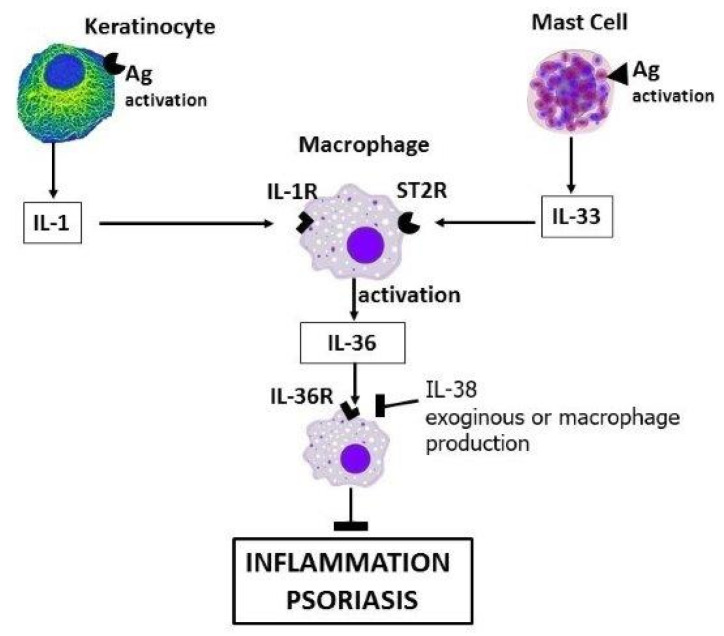
Keratinocytes and mast cells secrete proinflammatory cytokines IL-1 and IL-33, which activate macrophages to generate IL-36. The inhibitory effect of IL-38 through the IL-36 receptor binding leads to inhibition of inflammation in psoriasis.

It has been recently reported that IL-38 plays key role in many inflammatory disorders, including asthma, rheumatoid arthritis, and atherosclerosis [68]. IL-38 (or IL-1F10), the last cytokine member of the IL-1 family, was discovered in 2001 [52]. IL-38 binds to IL-36R6 (IL-36 receptor 6), causing anti-inflammatory activity by reducing several cytokines—including IL-1, IL-6, and IL-8—in experimental inflammatory diseases [69] (Figure 2). The IL-1 family includes two receptor antagonists (IL1Ra and IL-36Ra), two cytokines that suppress inflammation (IL-37 and IL-38), and seven proinflammatory cytokines, including IL-1 and IL-33. IL-38—also referred to as IL-1F10—is similar to the IL-1 receptor antagonist (IL-1Ra), and to the IL-36 receptor antagonist (IL-36Ra). IL-38 is expressed in many cells, but in particular in epithelial cells, B cells, and tonsils—all elements of defense against external invaders [69]. The cellular receptor of IL-36 specifically binds IL-38, and does not bind to IL1R1, IL-1R3, or IL-18Rα. By binding to the IL-36 receptor, IL-38 inhibits human mononuclear cells stimulated with IL-36 in vitro—an effect shared with IL-36Ra, which it resembles for its inhibitory effects, in particular on the Th-17 response [70]. In addition, human IL-38 inhibits the production of proinflammatory IL-17 and IL-22 by human peripheral blood mononuclear cells (PBMCs) in vitro [68]. In IL-38-deficient mice, Th17 response is increased along with inflammation. In psoriasis, as well as other autoimmune inflammatory diseases, IL-17 plays a role as a very important cytokine in the inflammatory process, and is a crucial key in the pathogenesis of various chronic proinflammatory diseases [70]. Therefore, IL-17—which strongly induces IL-36 in psoriasis—is a target molecule for the study of new therapies in autoimmune disorders. It should be emphasized that this inhibitory effect of IL-38 on the IL-36 receptor is almost always partial, and with different effects, as it depends on the cells or tissues examined. The cytokine IL-38 generates new hope in the treatment of diseases that are difficult to cure, such as chronic proinflammatory diseases, mediated by inflammatory cytokines of the IL-1 family.

The inhibition of inflammatory members of the IL-1 family has attracted much attention from researchers for the treatment of various inflammatory diseases, including psoriasis. Thus, here we can confirm that the cytokine IL-38 emerges as an inhibitor of inflammation, and is an important suppressor molecule and interesting marker in diseases [71]. However, the power of the anti-inflammatory effect of IL-38 still has some dark points, since the binding to its receptor is quite weak, and the effective concentration of this cytokine in humans is still unknown. Therefore, IL-38 holds promise of innovative therapeutic tools, but more in-depth studies are needed in order to establish the true biological value of this interesting novel cytokine.

## 8. Suppression of Inflammation Due to IL-37

MCs are activated during allergic and anaphylactic reactions by mediating innate and acquired immunity, but these cells can also be activated by non-allergic triggers such as neuropeptides and cytokines, as occurs in certain neurological diseases [72]. In fact, IL-1 has the ability to stimulate MCs to produce IL-6, TNF, and IL-33 without degranulation, building a powerful proinflammatory network—an effect that increases with stress, which exacerbates psoriasis. Therefore, targeting IL-1 could represent a very significant tool to inhibit inflammation. IL-37 is an important suppressor of innate immune cells, which could lead to new potential therapeutic approaches and applications. IL-37 is a member of the IL-1 gene family that broadly inhibits inflammation in various inflammatory and autoimmune diseases, while also reducing acquired immunity, paving the way for the healing of psoriasis [73]. In fact, the dendritic cells (DCs) that express IL-37 are tolerogenic—an effect that reverberates on the activation of the responses of effector T cells, inducing Treg cells and inhibiting adaptive immunity [74]. In rodent experiments, IL-37 has shown protection in many induced inflammatory pathologies, while also suppressing the immune response [75]. This new innate cytokine inhibitor non-specifically reduces several inflammatory cytokines and chemokines in autoimmune rheumatic diseases by acting on the suppression of mTor and increasing the activity of AMP kinase [76]. Therefore, administration of recombinant human IL-37 to rodents reduces inflammatory metabolism and increases oxidative phosphorylation by acting at both the systemic and local tissue levels, including in the skin. IL-37 inhibits the IL-1β, IL-6, and TNF cytokines—which play fundamental roles in psoriatic inflammation—but it can also suppress some chemokines, such as CCL2, which is also important in psoriasis and rheumatic diseases [77]. Levels of the IL-37 protein and its mRNA are lower in healthy humans, while individuals deficient in IL-37 may be more prone to inflammatory pathologies [78]. In fact, this cytokine was found to be higher in some rheumatic diseases, including psoriasis, showing that the body produces IL-37 as a response to excessive inflammation [79]. Several authors report that IL-37 is involved in rheumatic diseases and psoriasis, and could certainly play a role in the treatment of these complex pathologies [72,80,81,82].

In this article we report that IL-38 and IL-37 may represent a new potential anti-inflammatory therapeutic pathway in psoriasis, and probably in other skin diseases mediated by IL-1 family members. The inflammatory suppression in psoriasis can be carried out by these new cytokines, which can give a specific therapeutic aid compared to the classic therapies used today. Therefore, acting specifically on inhibiting inflammatory cytokines is much better than using non-specific inhibitory drugs that circulate throughout the body, even in unwanted places. In this article, we show for the first time the interrelationships between mast cells, cytokines IL-1, IL-33, and IL-36, and inflammatory states in psoriasis.

Therefore, IL-37, IL-38, and IL-1Ra fuel new therapeutic and innovative hopes [81], but more in-depth studies are needed to establish the true biological value of these interesting anti-inflammatory cytokines.

## Figures and Tables

**Table 1:** Mast cells: stored and synthesized compounds.

**Stored Enzymes**
Tryptase
Chymase
Cathepsin G
Phospholipase
Kininogenase
Carboxypeptidase A
Cholinesterase
**Stored Cytokine**
TNF
**De Novo Synthesized Cytokines**
Cytokines: IL-1, 2, 3, 5, 6, 7, 8, 9, 13, 16, 17, 33, and TNF
Arachidonic Acid product: PGD_2_

Mast cell activation with anti-IgE leads to mast cell degranulation, resulting in acute tissue swelling and local arrangement of fibrin, acute airway hyperresponsiveness (AHR), local recruitment of leukocytes into the skin and stomach wall, and cardiopulmonary changes.
